# Comprehensive Advancements in Hydrogel, and Its Application in Microalgae Cultivation and Wastewater Treatment

**DOI:** 10.4014/jmb.2407.07038

**Published:** 2024-10-31

**Authors:** Guangtao Yang, Jinglin Zhang, Rosazlin Abdullah, Wai Yan Cheah, Dehua Zhao, Tau Chuan Ling

**Affiliations:** 1 Institute of Biological Sciences, Faculty of Science, University of Malaya, 50603 Kuala Lumpur, Malaysia; 2 Graduate School of Life Sciences and Health, Faculté des Sciences, Université Paris-Saclay, 91400, Orsay, France; 3 Centre for Research in Development, Social and Environment (SEEDS), Faculty of Social Sciences and Humanities, Universiti Kebangsaan Malaysia, 43600 UKM Bangi, Selangor Darul Ehsan, Malaysia; 4 Department of Civil Engineering, Faculty of Engineering, Universiti Malaya, Kuala Lumpur 50603, Malaysia

**Keywords:** Microalgae, cultivation, hydrogel, wastewater treatment

## Abstract

Microalgae are recognized as a sustainable resource to produce biofertilizers, biofuels, and pigments, with the added benefits of environmental sustainability, such as carbon sequestration and pollutant removal. However, traditional cultivation methods face challenges like low biomass productivity and high operational costs. This review focuses on the innovative use of hydrogels as a medium for microalgae cultivation, which addresses these challenges by enhancing nutrient permeability, light distribution, and overall growth efficiency. Hydrogels provide a three-dimensional matrix that not only supports higher biomass yields but also facilitates the removal of pollutants from wastewater, contributing to circular economy goals. The review also explores the environmental benefits, challenges, and prospects of integrating hydrogel technology into microalgae cultivation systems. By highlighting influencing factors through which hydrogels improve microalgal productivity and environmental outcomes, this work aims to provide insights into the potential of hydrogel-based systems for sustainable development.

## Introduction

The rapid growth of the global population [1] has led to increased challenges [2, 3] such as food shortages [4], energy crises [5], and environmental degradation [6]. Traditional agricultural practices, including the excessive use of chemical fertilizers, have exacerbated soil degradation [1] and contributed to environmental pollution. For instance, in North China, heavy nitrogen input from chemical fertilizers [7] has resulted in soil acidity and increased salinity [8].

To address these challenges, the scientific community is increasingly turning to sustainable technologies, particularly the utilization of microalgae. Microalgae are considered as a promising resource for producing biofertilizers [9] and biofuels [10], offering environmental benefits such as carbon sequestration and pollution mitigation. Unlike conventional crops, microalgae do not require arable land and can be cultivated in various environments, making them an ideal candidate for sustainable agricultural [11] and energy solutions [10, 12-15].

Microalgae-based biofertilizers and biofuels not only meet the demand for these products but also contribute to achieving a circular economy and carbon neutrality . However, the large-scale cultivation of microalgae still faces several challenges, including high production costs, low productivity, and energy-intensive processes. Recently, hydrogel technology has emerged as a novel medium for microalgae cultivation to overcome these challenges, offering significant potential. Hydrogels provide a three-dimensional porous structure that supports efficient nutrient and light transport, thereby optimizing microalgal productivity [16].

This review focuses on the application of hydrogel technology in microalgae cultivation and its potential environmental benefits. The analysis highlights the critical role of hydrogels in advancing microalgae-based solutions for sustainable development. Moreover, the application of hydrogels in wastewater treatment has demonstrated significant environmental benefits. For instance, hydrogels can be used to recover nitrogen and phosphorus from wastewater, reducing the ecological problems caused by harmful algal blooms . This technology not only supports nutrient recovery from wastewater but also enhances microalgal biomass production, enabling effective wastewater treatment and resource recovery.

## Hydrogel Applications in the Microalgae Cultivation System

Hydrogels are commonly utilized as biomaterial because of their high air permeability [17]. Calcium alginate-based hydrogels have been identified as having optimal permeability for essential elements such as nutrients, light, and gases (O_2_ and CO_2_), vital for facilitating the growth of microalgae [18-20]. Moreover, incorporating hydrogel into the microalgae cultivation process [21] can improve space utilization [22], protect microalgae against contamination [23, 24], minimize water consumption and reduce environmental impact [25], enhance photosynthesis [26] and growth rates, and increase the yield of secondary metabolites [27].

On the other hand, the conventional cultivation of microalgae, along with their symbiotic interactions with bacteria in wastewater treatment poses various challenges. Physically separating the microalgae from the bacteria is difficult [28], but this is crucial for the efficient extraction of microalgae biomass for subsequent utilization and the prevention of nutrient-rich microalgae being consumed by other heterotrophic bacteria [23]. Secondly, microalgae may receive insufficient light irradiation in a murky cell suspension with bacteria [29]. Lastly, due to the inherent slower growth rate of microalgae compared to bacteria, it is difficult to operate bioreactors with continuously reduced hydraulic retention durations. Therefore, entrapping microalgae in a porous and light-transmitting matrix such as alginate offers high-quality and acceptable permeability, biocompatibility, and transparency, which ensures unhampered gas exchange and allows for continuous operation, as well as easier harvesting of microalgae from wastewater.

Currently, the immobilization of microalgae, which involves encapsulating living cells in a polymeric matrix [30] allows the permeation of a solution containing nutrients and other components. This technique has emerged as a valuable method for various environmental applications?including the effective removal of undesired substances from water, efficient management of culture collections for CO_2_ capture, advancement of biosensor technologies [31], and the development of sustainable energy sources, among others [24]. Hydrogel-based immobilized cultivation not only promotes a high growth rate of microalgae in limited space but also eliminates physical-chemical concentration processes, such as flocculation and coagulation. Methods of immobilizing microalgae include using a nanoporous silica matrix [32] and utilizing naturally occurring polysaccharides including agars, carrageenans, and alginates [33].

Significantly, the continuous provision of nutrients through the channels enables microalgae residing within the hydrogel to proliferate and grow under specific ambient conditions over an extended period. This mode of cultivation offers advantages in terms of metabolic bioactivity compared to conventional flat sheet in large solution volume [34]. The quantification of microalgae growth inside the alginate beads exhibited an exponential pattern under symbiotic conditions, indicating that the efficiency of the bioreactor is constrained by the O_2_ production rates. In one previous study, Calcium alginate-based hydrogels demonstrated exceptional permeability to nutrients, CO_2_ and O_2_ [19]. Furthermore, alginate can protect microalgae from native microorganisms [24]. In the co-culture system of *M. kaistiae* KAS603, O_2_ microsensor measurements indicated a 4-fold increment in net photosynthesis compared to monoculture [22]. Additionally, the chlorophyll concentration of immobilized microalgae exhibited an increase of more than 30% under symbiotic conditions [33]. Kimet *et al*. (2022) mixed the alginate solution with the microalgae culture solution and subsequently applied this mixture into the nanocomposite (NC) hydrogel. Within the alginate gel, *Raphidocelis subcapitata*, a living microalga, was immobilized. Observations revealed that the presence of the microalgae had no impact on the stability of the adhesion between two layers of gel (the alginate gel and the NC hydrogel). Additionally, a significant amount of chlorophyll was detected, and the alginate gel turned green because of the growth of immobilized microalgae in it after a period of ten days. Demonstrating the stable adherence to concrete, the interfacial adherent hydrogel presents promising potential for the applications in bio-immobilization and bio-repository purposes [35].

González-Delgado, *et al*. examined the polymerization process of hydrogels with the aim of generating a suitable nanoporous structure for the immobilization of microalgae, which was accomplished by using transient UV light exposed with an intensity range of 140 to 700 mW/cm^2^ for a duration of 0.8 h [25]. The results indicated that the transient light polymerization has a notable impact on the average pore size and its distribution within the hydrogels. In addition, the specific proportions of monomer, initiator, and solvent influence the properties of the hydrogels. Accordingly, it enables the adjustment of nanoporous morphology within hydrogels, thereby enhancing the attachment capability of microalgae with differing sizes and shapes. Moreover, this technique facilitates the development of improved hydrogels for novel applications centered on microalgae immobilization [24]. In terms of environmental applications of immobilized microalgae, one notable utilization lies in wastewater treatment. Critical considerations in this context include the utilization of organic materials and the removal of pollutants, which serve as essential nutrients for algae [24].

Table 1 presents various types of hydrogels used alongside their advantages and disadvantages. It could be seen that different hydrogels exhibit diverse applications based on their distinctive properties. Nanoporous silica matrix hydrogels, characterized by mechanical stability, are particularly suited for environmental use due to their selective pollutant transport capabilities. Alginate-based hydrogels, with excellent biocompatibility and absorbent characteristics, are being used in wound dressing and drug delivery, albeit exhibiting potential adhesion to wounds. Silk fibroin hydrogels, while biocompatible, are limited in their utility for complex medical scenarios. Agar hydrogels have great gelling properties but are susceptible to variations in production conditions. Carrageenan hydrogels, commonly utilized in the food industries, exhibit a reduced swelling capacity. Silk protein-based hydrogels demonstrate biocompatibility and versatile application potential. Poly (2-hydroxyethyl methacrylate)(PHEMA) hydrogels are acknowledged for their durability and suitable for diverse industrial uses. The specific properties of each hydrogel type determine its applicability in various fields.

## Advantages of Hydrogel in Microalgae Cultivation

### Improve Light Distribution within the Culture

During the cultivation process of microalgae, light is crucial for their growth. Fix microalgae in hydrogel, the adhesive surface has a stronger absorption of light. Therefore, light can be absorbed into the cultivation system. However, with the increase of the thickness of cultivation system, as light is emitted from top of the system, the reflectivity of light becomes stronger, and the permeability weakens. When the thickness of gel is 1 mm, the light transmittance of 2%, 4% and 6% (w/v) silk hydrogels measured at the maximum absorption wavelength (430 nm) of chlorophyll A in microalgae is higher than 60% [36]. The growth of microalgae under low layer of system were influenced, so Pierobon, *et al*. introduced a new approach of periodically harvesting microalgae biomass in hydrogel system, which enables long-term sustainability of biomass production [37].

In wastewater treatment and CO_2_ emission reduction, biofilm-based microalgae culture makes it an effective technology for high photosynthetic efficiency and density. Given their large accessible adsorption area, porous materials are ideal substrates for supporting microalgae biofilms. In Fu, *et al*. study, the silk/microalgae gels were found to sequester carbon at a rate of 3.59 mg/l/min [36]. However, it is challenging for the linked cells to perform photosynthesis due to mutual shadowing, which hinders the transfer of external light to the inner surfaces. To address this issue, Huang, *et al*. [38] introduced light-guided particles (SiO_2_) into a photosensitive resin, creating a precisely engineered light-guided porous photobioreactor (PBR) using advanced 3D printing technology. This innovation achieved a 13.6-fold increase in the effective adsorption area of the PBR and significantly improved its space utilization. Additionally, a thermos-responsive hydrogel was cleverly attached to the surface of the substrate to form an innovative interface that can regulate temperature and enhance the adsorption and desorption processes of microalgae in both directions. An impressive acceleration in the growth rate of microalgae, amounting to 3.572 g m^−2^ d^−1^, was achieved due to a 103% increase in the adsorption capacity of the microalgae cells. Furthermore, the biofilm's desorption capacity surged by 564.7%, demonstrating the significant impact of temperature control on the process of microalgae harvesting [38].

Cultures encapsulated in gel result in the formation of cell aggregates due to the mechanical confinement of the cells. These aggregates significantly influence the light management within gel-encapsulated photobioreactors, thereby strongly affecting photosynthetic efficiency. The heterogeneous distribution of cell aggregates in a hydrogel matrix can enhance light penetration and more effectively mitigate photoinhibition compared to a flat biofilm. Additionally, incorporating scattering particles into the hydrogel matrix can further enhance light harvesting efficiency, resulting in a fourfold increase in biomass growth [21]. Chua, *et al*. studied the growth of microalgae aggregates within the hydrogel through incorporating scattering particles into the hydrogel matrix. And this approach can improve light distribution and maximize biomass growth [21]. Additionally, photo-initiators such as 1-[4-(2-hydroxyethoxy)-phenyl]-2-hydroxy-2-methyl-1-propan-1-one (Irgacure 2959, I2959), lithium phenyl (2,4,6-trimethylbenzoyl) phosphinate (LAP), ruthenium (Ru), and sodium persulfate (SPS) can be incorporated into the hydrogel fabrication process. For example, gelatin-methacryloyl (Gel-MA) hydrogels cross-linked with Ru/SPS exhibit significantly better light penetration depth than those using the I2959 system. These Ru/SPS cross-linked hydrogels also have higher glycosaminoglycan content and re-differentiation capacity, while exerting a less detrimental impact on the viability and metabolic activity of encapsulated human articular chondrocytes over a period of up to 35 days [39].

### Increasing Microalgae Productivity

Cultivating microalgae with hydrogels can greatly improve their productivity. Gorin, *et al*. examined the productivity of the microalgae species *C. vulgaris* and found that it exhibited a remarkable increase of 53.4% when subjected to adaptive illumination and cultivated in a hydrogel medium enriched with nanoparticles [40]. Pierobon, *et al*. evaluated how the frequency of harvesting affects the biomass production of high-density surface layers [37]. Their data indicated that in hydrogels made with 150 mM Ca^2+^, the first harvested layer consistently had the highest individual layer productivity, reaching a maximum of 1.0 g_DW_ m^-2^ d^-1^ in hydrogels with a 3-day harvesting period. For *Synechococcus elongatus*, productivity was also higher comparable to suspension cultures supplemented with high carbon concentrations under identical lighting conditions [37]. Regarding biomass yields, the calcium ion content also has an impact on biomass yield. Hydrogels made with 150 mM Ca^2+^ produced considerably more biomass during the first harvest time compared to those made with 400 mM Ca^2+^. About 0.11 g_DW_ m^-2^ d^-1^ of 150 mM and 0.04 g_DW_ m^-2^ d^-1^ of 400 mM were harvested over a period of two days.

Hydrogel culture increases the growth rate of microalgae. Krujatz, *et al*. investigated the growth *Chlamydomonas reinhardtii* 11.32b and **Chlorella* sorokiniana* UTEX1230, both immobilized in 3D-plotted hydrogel structures and suspension cultures under identical temperature and lighting conditions [41]. At all selected temperatures (26°C, 30°C, 37°C) and under both continuous illumination and 14/10h light/dark cycles, the growth rates of *C. reinhardtii* 11.32b immobilized in hydrogel remained stable between 0.4 and 0.6 d^-1^. In contrast, the growth rates of suspension cultures declined as the temperature increased under continuous lighting. And at 37°C with a 14/10 h light/dark cycle of illumination, hydrogel-embedded cultures exhibited a 44% higher growth rate compared to suspension cultures [41].

The thickness of gels also influences the growth rate of microalgae. Yuhang Fu tested the effect of varying gel thickness (ranging from 1 to 3 mm) on microalgae activity, using a silk fibroin concentration of 6% and an initial microalgae density of 1 × 10^3^ cells/ml. The microalgae growth rates in the three gel samples were comparable during the initial two days. However, from days three to seven, microalgae in the 1 mm thick gel grew more rapidly compared to those in the 2 mm and 3 mm gels. On day 7, the density of microalgae in sonicated gels with a thickness of 1 mm reached 5.28 × 10^5^ cells/ml, whereas it was only 4.12 × 10^5^ and 3.98 × 10^5^ cells/ml for gels with a thickness of 2 and 3 mm, respectively [36]. Likewise, Pierobon, *et al*. found that hydrogel cultures with the lesser thickness exhibited higher specific growth rates, with 1.0 mm hydrogels achieving rates of 0.28 ± 0.05 day^-1^ and 4.0 mm hydrogels reaching a maximum of 0.18 ± 0.03 day^-1^[37]. Lastly, the initial microalgal seeding density in hydrogel influences the growth rate of microalgae. Fu, *et al*. indicated low-density microalgae had a faster proliferation rate than the high-density microalgae in the gels [36].

Table 2 highlights the increased productivity of microalgae cultivated in hydrogel mediums. For instance, the cultivation of *C. vulgaris* in a hydrogel medium enriched with nanoparticles showed an increase of 53.4% in productivity. Likewise, thinner hydrogel cultures (1.0 mm) achieved higher specific growth rates (0.28 ± 0.05 day^-1^) compared to thicker ones (4.0 mm). These findings suggest that using hydrogels in medium can significantly enhance microalgae growth.

### Immediate Release of Oxygen and Increased Hydrogen Production

The oxygen production efficiency in microalgae-embedded hydrogels is higher than those suspended in solution where microalgae are not immobilized. Fu, *et al*. investigated the emission of O_2_ within a silk/hydrogel system, which involved applying a thin layer (1 mm thick) of the microalgae/silk hydrogel onto the surface of a dialysis tube. Silk hydrogel with microalgae produces 6.13 mg/l oxygen for 7 consecutive days under high density (7.6 × 10^5^ and 7.8 × 10^7^ cells/ml). When placed in a sealed flask containing CO_2_ gas, the system consistently generates oxygen (151 ml) for a minimum of 60 days. The efficiency of oxygen generation in this system is six times more than that of the control microalgae suspension culture [36]. O_2_ plays a crucial role in every stage of the wound-healing process. Corrales-Orovio, *et al*. incorporated *Chlamydomonas reinhardtii* into alginate hydrogels to produce O_2_ [42]. This integration resulted in efficient release of O_2_ and bioactive compounds when exposed to light. These photosynthetic hydrogels present a viable and effective approach for facilitating wound healing by providing a localized source of O_2_ and other bioactive compounds.

For hydrogen (H) production, immobilized cell systems exhibit a distinctive advantage over conventional cell suspension methods. Das, *et al*. created a completely biomimetic leaf-shaped device for hydrogen production device that allows the combined fabric to fix microalgae and culture medium to hydrate simultaneously, and harvest from the generated H_2_ in a continuous flow state without requiring replacement of the algae culture [43]. The hydrogen production per gram of algae using artificial leaf devices is 20 times higher than previously reported batch reactors. In comparison to batch reactor, which produced H at a rate of around 0.082 mL per gram of cell, the device produced H at a rate of about 0.794 mL per gram of cell.

## Encapsulating Microalgae in Hydrogel to Treat Wastewater

### Recovery of Chemical Elements from Wastewaters

The excessive use of chemical materials has resulted in elevated concentrations of chemical elements in wastewater. To address this issue, microalgae can be cultivated in wastewater to utilize these elements, such as P and N, which are required for microalgae growth [44]. Further, in hydrogel system, microalgae are as well able to assimilate these elements effectively from wastewater. As shown in Table 3, Xiao, *et al*. developed hydrogel composites synthesized with calcium phosphate and wollastonite particles, which effectively adsorbed P and subsequently precipitated as calcium phosphate [45]. This process not only mitigates harmful algal blooms (HABs) but also reclaim the P that fuels algal growth [45]. The use of alginate for immobilizing microalgal cells also present a promising solution for the remediation of contaminated rivers, as it has proven effective in removing contaminants such as palm oil, ammonium (NH_4_), and other pollutants [46].

When microalgae were encapsulated together with bacteria in hydrogel, the removal efficiency increased significantly. *C. vulgaris* was encapsulated in alginate beads by Praveen and Loh [33] and introduced into a bioreactor along with *Pseudomonas putida* for the treatment of synthetic wastewater. When immobilized microalgae were introduced into the system during continuous operation, the removal efficiency at a glucose concentration of 500 mg/L increased significantly from 73% without aeration to complete removal, reaching 100% [33]. The symbiosis of microalgae-bacteria has been accelerating wastewater remediation. However, the concentration of medium of symbiosis between could affect the removal of chemicals. Research by Cai, *et al*. [47] demonstrated that sludge beads with a concentration of 30 g/l exhibited excellent performance, achieving a chemical oxygen demand (COD) removal rate of 84.8%, which fell slightly below the desired threshold of 90%. By introducing the microalgae *C. vulgaris*, a consortium of microalgae-activated sludge was created inside the immobilized beads. This led to improved performance in breaking the ceiling effect, with a COD removal rate above 90%. The ability to remove COD might be enhanced (with a COD removal rate above 92%) by using an LED light belt [47]. The N removal using hybrid hydrogels that contained a consortium of microalgae and nitrifying bacteria was different, when activated carbon (AC) was included as an adsorbent for inhibitory substances. For hydrogels without AC, final concentrations of NH_4_/L for hydrogels were 3.13 mg and 3.75 mg at 72 h with 5% and 16% of the consortium biomass, respectively. While at 72 h, the NH_4_ concentration in hydrogels without AC was around 14 mg/l, indicating higher NH_4_ removal efficacy compared to hydrogels with AC. These results substantiated that hydrogels can perform the nitrification process independently, without the need for AC to remove potential inhibitors [48]. However, another study demonstrated that the presence of perfluorodecanoic acid (PFDA) did indeed influence the nitrification process [49]. PO_4_ was primarily eliminated using hydrogels containing AC, rather than hydrocarbon (HC, at 37.5%) or a combination of microalgae-bacteria (HB) and HC (HBC, at 29.2%). Conversely, NH_4_–N was removed by all types of hydrogels, with removal rate ranging from 61%to 79%. Furthermore, soluble algal products and polyvinyl alcohol (PVA) leaching from hydrogels contributed to an increase in COD.

The nitrification performance of the consortia was influenced by illumination conditions immobilizing consortia of *C. sorokiniana* and nitrifying bacteria within a light-shielding hydrogel matrix. The experimental settings for the nitrifying bacteria included using a light-shielding hydrogel with only nitrifying bacteria, excluding carbon black, and scattered nitrifiers without immobilization. The dispersion resulted in a significant reduction in nitrification activity and process failure at 1,600 μmol photons m^−2^s^−1^. The nitrification rates for the light-shielding hydrogel were about 9 and 2 times higher than those for the hydrogel non-light-shielding and dispersed conditions, respectively, resulting in complete nitrification with no nitrite buildup [50]. The sensitivity of microalgae-nitrifying bacteria to light can lead to less effective nitrogen removal or even complete failure in some cases. To address this, Nishi, *et al*. created a light-shielding hydrogel that effectively encloses nitrifying bacteria, promoting the development of a light-resistant consortium of microalgae and nitrifying bacteria [51]. Under circumstances of high illumination, this consortium has demonstrated effective ammonia removal.

Improved hydrogel materials have higher ion adsorption capacity. Hydrogels made from extracellular polymeric substances (EPS) and modified with iron (Fe) were used to recover phosphorus. The hydrogels were composed of agarose (AG) and agarose-humic (AH). The AG and AH hydrogels exhibited adsorption capacities of 33.9 and 67.7 mg P g^−1^, respectively. Both hydrogels, in their original form, exhibited an adsorption capacity of above 75% at the pH circumstances studied (pH 3−10) [52]. And in these modified-hydrogels, the presence of coexisting anions (*i.e.*, SO_4_^2-^, NO_3_^-^, and CO_3_^2-^) did not have a major impact on the adsorption performance, except for CO_3_^2-^, which hindered the process. The inclusion of competing ions in alginate-like exopolymers (ALE) hydrogels did not affect the removal of phosphate [53].

Moreover, pH values and viscosity affect the chemicals removal. The phosphate removal was significantly hindered by pH values that ranged from neutral to acidic (28.9 ± 0.8% at pH = 6.00). However, a basic pH of around 8.50 was found to be the most advantageous, resulting in a phosphate removal rate of up to 90.8% at pH = 8.67 [53]. Therefore, by closely monitoring and regulating external parameters such as pH, temperature, and salinity, the removal process may be enhanced while working with existing technology [54]. Also, biopolymers promoted the cells for further reuse and treatment and protected cells from toxic environment [54]. Moreover, the pH, alkalinity, and salinity of the reverse osmosis concentrate (ROC) have a collective influence on the physical stability, chemical properties, biomass production, and nutrient removal efficiency. The alginate beads containing *C. vulgaris* demonstrated high stability and effectiveness in removing nutrients (achieving up to 100% removal of TP and 85% removal of TN per treatment cycle lasting 48 h over a period of 10 days). Additionally, these beads were able to produce biomass at a rate of up to 340 mg/l/d. These results were observed when the salinity, pH, and alkalinity levels were below 8 g TDS/L, between 7 and 9.5, and below 400 mg/l, respectively. [55]. Chitosan and konjac flour with different viscosities can alter the durability of hardened gels during tertiary wastewater treatment processes involving high concentrations of phosphate salts (1 M Na_3_PO_4_). This chelating agent proved to be the most effective for cross-linking, while still allowing the diffusion of inorganic nutrients through the gels and not impeding the growth of microalgae in the chitosan-immobilized cell network. Immobilized cultivation based on high viscosity chitosan gels at a concentration of 2% (m/v) demonstrated greater chemical stability in a medium with 1 M Na_3_PO_4_ containing viable cells compared to low viscosity chitosan or mixed gels. Following the second uptake, NH_4_^+^-N and PO_4_3−-P were eliminated within 3 h. After the fourth uptake, nitrogen removal reached 99% within 2 hours, while phosphorus removal remained at 100% with the same incubation period [56].

Different types of microalgae have different ion absorption efficiency. Immobilized microalgae reached a removal efficiency of up to 60% (*C. vulgaris*) and 42% (*S. armatus*). Powdered zeolite was added in immobilized microalgae hydrogels that could release previously adsorbed ammonium nitrogen for uptake by microalgae. *C. vulgaris* and *S. armatus* immobilized with zeolites, demonstrated enhanced removal efficiencies, achieving rates of up to 86% and 79%, respectively [57]. In addition, utilize diatom synthetic substances to absorb and remove ions. Marine diatoms *Chaetoceros* sp. and *Thalassiosira* sp. can synthesize silver nanoparticles (AgNPs). Then enclosed diatoms in a Ca-alginate hydrogel bead and used *Thalassiosira* sp. mediated AgNPs to successfully eliminate phosphate and nitrate, achieving removal rates of 74% and 65% respectively. The Ca-alginate hydrogel beads showed notable removal rates: 64% for nitrate, 91% for phosphate, and 78% for COD. In contrast, the presence of *Chaetoceros* sp. resulted in a considerable reduction in COD by 73% via the mediation of AgNPs [58].

### Higher Removal Efficiency of Pollutants

Human activities have led to the indiscriminate discharge of a wide range of pollutants into extensive aquatic ecosystems, including rivers, seas, and oceans. Alginate, a biopolymer derived from brown algae, has emerged as a promising material for pollution mitigation. Its inherent structural composition, physical attributes, widespread accessibility, compatibility with biological systems, and biodegradability confer it with notable adsorptive properties. Consequently, these materials, including hydrogels, composites, and nanocomposites, have demonstrated potential in effectively removing methyl violet dye and heavy metals through various mechanisms. The immobilization of microalgal cells within an alginate matrix has shown superior capacity in removing pollutants from wastewater, surpassing the effectiveness of freely suspended microalgal cells and alginate alone. Based on the findings of Rafiee, it has been postulated that alginate possesses the potential to serve as a benign agent for eliminating contaminants from the environment [46].

*Chlorella*-bacterial cellulose (BC), known for its high-water content and biodegradability, is produced by *Gluconacetobacter hansenii*. These hydrogels can be utilized as an ethylene scavenger. Lee, *et al*. immobilized *C. vulgaris* in BC-based hydrogels, which were prepared using a combination of 2.4% carboxymethyl cellulose (CMC) and 1% agar [59]. This composition helps to maintain the temperature of the algae without the need for daily supply. And results showed a 90% reduction in ethylene levels. In Sole and Matamoros’ study, both the reactor for free and immobilized cultivation of microalgae can remove up to 80% of endocrine disrupting compounds within 10 days [60]. Fixing microalgae in alginate beads also improved the kinetic removal rates of bisphenol AF, bisphenol F, and 2,4-dichlorophenol. Although immobilized *C. vulgaris* alginate beads have been shown to have the potential to treat urban wastewater ROC, these conditions (pH, alkalinity, and salinity combinations) may not be conducive to the long-term use of alginate beads in ROC treatment. The empirical model developed and validated by Mohseni, *et al*. only requires input of pH, alkalinity, and salinity parameters, which can provide important information for technical decision-making, process optimization, and evaluation of process limitations, and improve the performance of alginate immobilized algal beads in treating ROC [55].

For the removal of heavy metals from wastewater, Leon-Vaz, *et al*. used copolymers to adsorb heavy metals from water, and phytoremediation with microalgae can increase the adsorption of metals (Cd^2+^, Cu^2+^) [61]. A comparison was made between the use of copolymers and microalgae alone, as well as immobilization (AlgaPol) for metal adsorption. The results showed that AlgaPol biofilm was able to remove more than 90% of these metals from the growth medium.

Table 4 illustrates the effectiveness of hydrogels in pollutant removal. For instance, CMC-Fe_3_O_4_ hydrogels showed a maximum adsorption capacity of 600 mg/g for methylene blue and 100 mg/g for CdCl_2_, indicating their high efficiency in removing pollutants from water. These findings underlined the potential of hydrogels as effective materials for environmental remediation.

## Future Perspectives and Conclusion

The cultivation of microalgae within hydrogels and encapsulating microalgae in hydrogels for wastewater treatment represent burgeoning areas of microalgal research. In this review, a multitude of benefits of hydrogel-based microalgae cultivation have been summarized, exhibiting its considerable values as a viable alternative of conventional cultivation and advantages of this cultivating mode in wastewater treatment, compared with non-hydrogel. The integration of hydrogels in microalgae cultivation presents a host of opportunities to enhance productivity, optimize resource use, and expand applications. Here are key future perspectives to consider:

• Optimizing Light Distribution: Hydrogels can be engineered to improve light penetration and distribution within microalgae cultures, thereby increasing photosynthetic efficiency.

• Enhanced Nutrient Delivery: Hydrogels can serve as a medium for delivering nutrients directly to microalgae, ensuring optimal growth conditions and potentially reducing the need for external fertilization.

• Development of Smart Hydrogels: The advancement of responsive or "smart" hydrogels that can adapt to environmental changes could significantly improve the efficiency and adaptability of microalgae cultivation systems.

•Wastewater Treatment Applications: By encapsulating microalgae within hydrogels, the efficiency of nutrient and pollutant removal from wastewater can be significantly improved, creating a sustainable cycle of resource recovery.

• Agricultural Use: Hydrogel-based microalgae composites can be used as soil conditioners to enhance water and nutrient retention, providing a slow-release fertilizer effect and promoting plant growth.

• Bioenergy Production: Harnessing microalgae cultivated in hydrogels to produce biofuels, particularly hydrogen, presents a sustainable alternative to fossil fuels, given microalgaés rapid growth and CO_2_ sequestration capabilities.

• Environmental Remediation: Hydrogel-based microalgae systems have shown high efficiency in removing organic and inorganic pollutants from water, suggesting potential for broader environmental applications.

• Addressing Commercialization Challenges: While hydrogel-based cultivation offers many advantages, challenges such as light limitation in high-density cultures need to be addressed. Future research should focus on strategies to overcome these barriers, facilitating the large-scale commercial application of this technology.

By pursuing these directions, the future of microalgae cultivation using hydrogels may lead to more sustainable practices, higher yields, and a broader impact on various sectors, from agriculture to energy production.

## Figures and Tables

**Table 1:** Characteristics of hydrogels with their advantages and disadvantages.

Types of hydrogels	Advantages	Disadvantages	References
Nanoporous silica	Mechanically stable; resistant to degradation by microorganisms; porosity can be adjusted by manipulating the synthesis parameters enabling the unhindered diffusion of high molecular weight molecules; controlled porosity and the potential of silica surface derivatization allowing for selectively transport specific pollutants, imparting distinct selectivity to each module	The controlled porosity and ability to modify the surface of the material with silica allow for the targeted transport of specific contaminants, thereby giving each module in the configuration its own unique selectivity.	[32]
Alginate-based hydrogels	Good biocompatibility and liquid absorption capacity. be used in wound dressing, tissue engineering, and drug delivery applications.	The risk of harm exists when removing the dried hydrogel or alginate because it adheres to the wound bed; alginate has a tendency to accumulate the exudate emanating from the wound and disperse it towards the intact skin adjacent to the lesion.	[62]
Silk fibroin hydrogelsmatrix	Safety attributes, biocompatibility, regulate the process of degradation, and capacity to seamlessly integrate with other materials	The current state of functional silk fibroin hydrogels is insufficient to meet the requirements of complex application scenarios.	[63]
Agars	Excellent gelling properties; form relatively high thermal stability and strength gels.	The implementation of rigorous conditions during the alkali pre-treatment led to the partial degradation of the agar fraction, leading to a reduction in molecular weight in comparison to the unpurified extracts; In regards to the agar extraction procedure, the implementation of sonication yielded extracts that exhibited reduced agar contents and molecular weights.	[64]
Carrageenans	Adding κ-carrageenan to the bigel system will increase hardness and decrease cohesiveness.	Lower swelling capacity	[65]
Silk protein-based hydrogel	Biocompatibility and ambient conditions gelation; silk--- formidable mechanical characteristics, safe and compatible nature, green sourcing, and v versatility in terms of materials formation. long-term cell vitality and function		[66]
PHEMA hydrogels	Highly durable; maintaining their structural integrity over an extended period of time when exposed to a liquid; PHEMA disks---altering porosities through the manipulation of the polymerization mixturés composition, modifiable surface properties by facilely attaching a diverse array of synthetic or natural molecules onto their surfaces		[67]

**Table 2:** High growth of microalgae cultivated in hydrogel system.

Hydrogel	Microalgae	Cultivation	Performance	Reference
Gelatin	*Marinichlorella kaistiae* KAS603	Co-cultivate with bacteria (Erythrobacter sp)	3-fold enhancement of growth compared with monoculture	[22]
Gelatin-modified poly 2-hydroxyethyl methacrylate (PHEMA)	*Nannochloropsis* sp., *Dunaliella salina* and *Botryococcus braunii*	Immobilization and addition of crosslinking agnet	5-20 times higher photosynthetic activity than freshwater microalgae	[67]
Calcium alginate	*Synechococcus elongatus* PCC 7942	Immobilization	Achieved 0.08-3.1 × 10^9^ cells/ml production densities comparable to that of biofilms	[37]

**Table 3:** The applications of microalgae-based hydrogels in chemicals removal from wastewaters.

Hydrogel	Concentration	Conditions	Types of microalgae	Targeted Chemicals	Efficiency	Advantages	Disadvantages	References
Mineral-hydrogel composites (calcium phosphate and wollastonite particles)	Dry weight: 36% alginate, 48% CaP mineral seed, and 16% wollastonite; 2.3% CaP, 1.7% alginate, and 0.8% wollastonite in water content.	Municipal wastewater agricultural run-off	*Synechococcus elongatus 2973*	P→Ca_3_(PO_4_)_2_	Removed 96% Removed 91%	A new approach for capturing P using heterogeneou s mineral nucleation and growth	-	[45]
Na-alginate	2%	Wastewater	*Chlorella vulgaris*	Total N Total P	Food grade (FG): 42 ± 3, 59 ± 4 Low viscosity (LV): 42 ± 5, 60 ± 1 Medium viscosity (MV): 43 ± 8, 58 ± 1 Wastewater control: −22 ± 37, 26 ± 3 FG: 20 ± 2, 44 ± 11 LV: 20 ± 9, 50 ± 13 MV: 21 ± 6, 55 ± 13 Wastewater control: 4.7 ± 12, −5.9 ± 14	LV and MV alginate protect cells from physical damage in wastewater	LV and MV alginate restrict nutrients diffusion; FG alginate has highest transmittance, leading greater light and inhibiting cells	[68]
Extracellula r polymeric substances, Fe-modified hydrogels	Un-modified agarose (AG) hydrogel: 1%, 3%, and 5% un-modified agarosehumic (AH) hydrogel: (SH): AG), 0.1:1, 0.5:1, 1:1, 2:1, and 5:1 (sodium humate	Sewage	Microcystis	P	AG and AH hydrogels: 33.9 and 67.7 mgPg^−1^, respectively	Adaptable to wide-range pH values (3−10)	-	[52]
Alginate beads	2-4% (w/v) Sodium alginate	Synthetic wastewater	*Chlorella vulgaris* & *Pseudomonas putida*	Glucose	From 73% without aeration to 100% removal	Gas exchange between suspended bacteria and immobilized algae	-	[33]
Carbon black-added alginate	2.0 wt% Sodium alginate, 0.2 wt% carbon black particles	-	*Chlorella sorokiniana*	NH_4_ NO_3_ NO_2_	1600 μmol Photons: 100% nitrification	Light-shielding properties enhanced nitrification and ammonia removal	-	[50]
Sodium alginate	2%	Acid mine drainage (AMD)	*Desmodesmus* sp. MAS1 and *Heterochlorella* sp. MAS3 (acid-adapted strains)	Fe	80% (in 24 h) 68% (in 24 h)	>1.20–1.50- fold with alginate-beads	Put dash in column without data or info	[19]
Polyvinyl alcohol (PVA) & sodium alginate (SA)	10 wt% PVA, 2 wt% SA	Deionized water Initial ammonium concentration 77 ± 0.5 mg/l	*Chlorella vulgaris* & nitrifying bacteria	Ammonium	24 h: 28.47 and 23.80 mg/l for 5%, 16% biomass (without activated carbon, AC) 48 h: 21.93 mg/l for 5% and 16.19 mg/l for 16% (without AC)			[48]

**Table 4:** Pollutants removal by treatment of microalgae-based hydrogel system.

Hydrogel	Microalgae	Pollutants	Performance	Assessment	Reference
CMC-Fe_3_O_4_		Methylene blue (MB) CdCl_2_	Maximum amount: 600 mg/g dried hydrogel; q_m_ = 620 ± 100 mg/g, K_L_ = 0.8 ± 0.1 q_m_ =100 ± 10, K_L_ = 0.06 ± 0.01	Eco-safety High adsorption of MB and Cd	[69]
Biochar and chitosan (BC)		Ciprofloxacin (CIP) enrofloxacin (ENR)	pH = 7 1.0 g/l BC, 300min 1.5 g/l BC, 450 min	A low-cost adsorbent	[70]
Nanocellulose from	*Chlorella vulgaris*	Methylene blue (MB)	Add microalgae Nanocellulose into CMC/SA, adsorption capacity: 28.57 mg/g Dye removal: 24.4%; CMC/SA/ 60AH: 109.03 mg/g	Showed similar characteristics and adsorption capacity with commercial Nanocellulose	[71]
